# 3D Object Detection Based on Polar Representation for Better Comprehensive Performances

**DOI:** 10.3390/s26165243

**Published:** 2026-08-19

**Authors:** Feng Gao, Jiaxin Chen, Niuniu Wang

**Affiliations:** College of Mechanical and Vehicle Engineering, Chongqing University, Chongqing 400044, China; 202307131198t@stu.cqu.edu.cn (J.C.); 202407131230t@stu.cqu.edu.cn (N.W.)

**Keywords:** autonomous driving, 3D object detection, multi-modal fusion, polar BEV

## Abstract

Multi-modal 3D object detection is an important task in autonomous driving systems, where cameras and LiDAR provide complementary semantic and geometric information. Most existing BEV fusion methods are designed based on the Cartesian representation space, which does not fully match the sensing geometry of camera and LiDAR. This generally leads to redundant computation in distant regions. To address this issue, GARF, a geometry-aware polar BEV framework, is presented for multi-modal 3D object detection. GARF organizes camera and LiDAR features in a unified polar BEV space, which can represent spatial resolution more compactly. For the camera branch, the uncertainty-guided transformation of the polar view is designed to improve the reliability of depth estimation. Then, the generated polar BEV feature is further refined to attenuate radial noise and angular discontinuity. For the LiDAR branch, the polar-aware sparse feature extraction and distortion correction modules are designed to deal with the anisotropic structure and geometric distortion caused by polar voxelization. For multi-modal fusion, the region-aware cross-modal fusion strategy and polar detection head with anisotropic Gaussian center response map are developed, which achieve effective feature interaction and consistent geometry supervision. The experimental results on nuScenes show that GARF achieves 71.8% mAP and 73.7% NDS, improving the baseline by 3.3% mAP and 2.3% NDS. Meanwhile, the inference speed increases from 7.1 FPS to 8.9 FPS, and the consumption of GPU memory decreases from 41,114 MiB to 33,346 MiB.

## 1. Introduction

Reliable 3D object detection is important for automated driving systems, since it needs to know information about surrounding objects such as category, position, size, orientation, and motion state [1]. Considering the fact that cameras and LiDAR can provide rich texture and spatial information, respectively, it becomes an effective and widely used way to achieve 3D object detection by combining them together [2,3]. Accordingly, how to fuse these two totally different types of information effectively in a unified space becomes a key problem. With the development of automated driving technologies and applications, the technical approach gradually converges to bird’s-eye view (BEV) representation, which transforms multi-view image and point cloud features to the top view [4,5]. BEV representation can provide a definite space for fusion and spatial alignment of different features, which is beneficial to the design of downstream tasks, such as motion planning. Benefiting from this, the existing methods have achieved strong performances on large-scale datasets [6,7,8,9,10,11]. Currently, most BEV fusion methods are built on Cartesian grids, where the physical space is uniformly divided along the x and y axes. This division method is straightforward and easy to understand, but it does not match the sensing patterns of camera and LiDAR closely. In fact, both LiDAR points and image pixels are dense in the near region and sparse in the far region [12,13]. As a result, in the distant region, there easily exist redundant computations for the detection and fusion models designed based on these uniform Cartesian grids. On the contrary, they may ignore some details of objects in the near region because of over-compression of sensor information.

**Figure 1:**
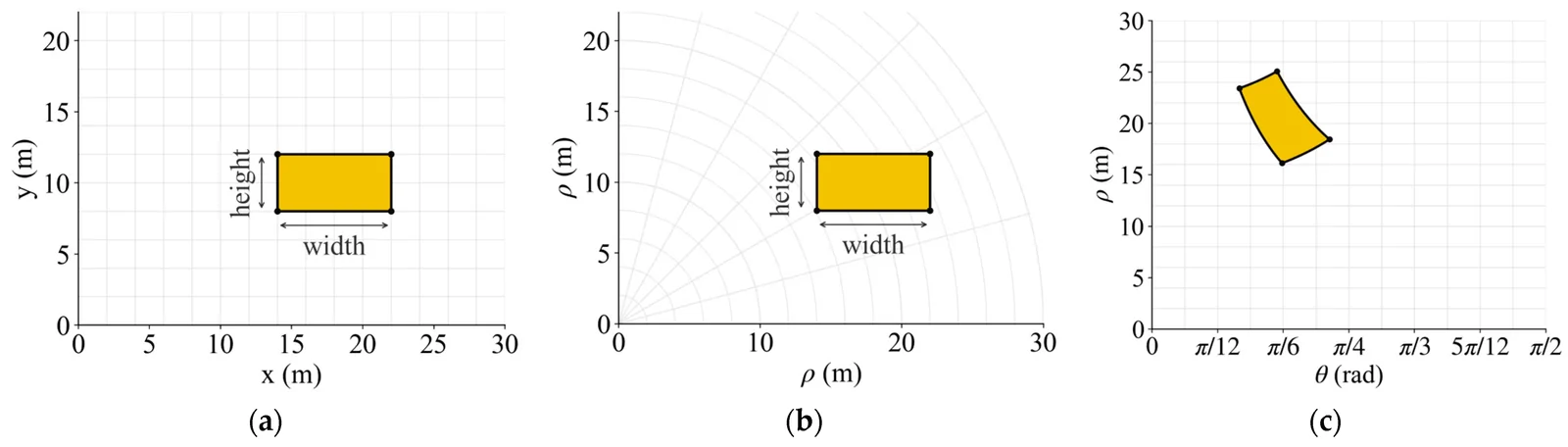
Object representation in different spaces: (**a**) Cartesian BEV; (**b**) polar BEV in physical space; (**c**) data space sampled by polar BEV.

As shown in Figure 1a,b, compared with the Cartesian grids, polar BEV representation is more consistent with the measurement mechanism of camera and LiDAR [14,15]. It divides the horizontal space into the angular and radial dimensions, which can match the radial scanning of LiDAR and the perspective projection of cameras better. In this way, the near region can be sampled more densely, while the far region is sampled more sparsely. Meanwhile, it aligns with human cognition better, i.e., more attention is paid to nearby objects. But unfortunately, other challenges are brought by the periodicity in the angular direction and the non-uniform scales in the radial direction. As shown in Figure 1c, these factors distort the object’s outline in the data space sampled by polar BEV. Compared with polar BEV, there is only scaling of the outline without distortion between the data and physical space of Cartesian BEV.

The distortion of the object outline brings new challenges to feature extraction, cross-modal fusion, object classification, and 3D bounding box prediction. To address these problems, we propose a geometry-aware 3D object detection framework (denoted by GARF) based on the polar representation of BEV to utilize the spatial distribution characteristic of original measured data, i.e., dense in the near region and sparse in the far region, for better comprehensive detection performance. The main contributions of this study are as follows:

1. In the backbone of the camera process branch, a geometry-aware feature model is presented to reduce the negative influence of depth estimation errors and view transformation uncertainty by enhancing the representation of BEV features, especially for the nearby objects, whose images have richer texture information;
2. In the LiDAR process branch, a feature correction module is designed to extract the polar voxel features in the presence of spatial distortion by considering the non-uniform distribution of polar grids, and accordingly, a more stable representation is achieved across different distance ranges;
3. In the fusion stage, an adaptive cross-modal module is designed to enhance the interaction between texture and geometric features to make better use of their complementary information;
4. Finally, a polar parameterized detection head with an anisotropic Gaussian center response map is presented to attenuate the geometric deformation of polar BEV between physical and data spaces, and accurately predict the center, offset, orientation, etc., of surrounding objects.

### 1.2. Related Works

According to the basic coordinate, main 3D object detection methods can be divided into two types, which are discussed and summarized as follows.

#### 1.2.1. Cartesian Coordinate

Since LiDAR measures the geometric information of objects finely, in the earlier stage most 3D detection methods were designed based on LiDAR. Several schemes, such as point [16,17], voxel [18,19,20], and pillar [21,22,23] have been proposed to divide the unstructured point clouds, which are further projected onto a regular BEV feature map, to extract and optimize the features. But LiDAR points lack texture information; they are not good at the classification of objects. On the other hand, vision-based 3D object detection using multi-view images has been widely studied recently. The typical LSS [13] based methods [24,25,26,27] generally predict depth distributions and then project image features into BEV through camera geometry. To aggregate related 2D semantics and establish the relationship between 3D and 2D, Transformer [28] based methods [4,29,30,31,32] use BEV queries to interact with image features with multiple views.

Although 3D object detection methods with single modality have made great progress, cameras and LiDAR still have some essential and complementary characteristics, such as depth uncertainty, illumination sensitivity, sparse point clouds, and limited semantic information. To address these issues, TransFusion [6] and BEVFusion [7] fuse camera and LiDAR features in a unified BEV space to improve the accuracy and robustness of 3D object detection. Besides camera and LiDAR, radar is further integrated to improve the robustness of detection results in complex conditions, especially adverse weather [33,34,35]. With the increase in information modes, some techniques have also been proposed to enhance feature interaction and alignment in BEV space [36,37,38]. Recent studies about geometry-aware perception and intelligent sensor fusion suggest that robust 3D perception should consider not only multi-modal feature aggregation, but also observation geometry, spatial reliability, and sensing characteristics of different modalities [39,40,41,42]. These provide useful motivation for the design of fusion representation with geometric consistency in this study.

#### 1.2.2. Polar Coordinate

Intuitively, polar coordinates better match the radial sensing geometry and non-uniform data distribution of the common environment sensors. Early studies have demonstrated that polar and cylindrical representations outperform Cartesian voxel-based methods in point cloud segmentation [12,14,43]. Inspired by this, polar coordinate-based object detection has attracted increasing attention and has been adopted in BEV representation recently. These studies show that polar space can reduce spatial redundancy in distant regions and improve the consistency between feature representation and sensor observation geometry from LiDAR [44,45] or camera [15,46,47,48]. These studies further find that the representation space itself affects feature organization and consistency of cross-modals, which motivates the investigation of polar BEV representation for fusion of camera and LiDAR in this study.

Table 1 compares the proposed polar BEV and other geometry-aware 3D detection methods. Some of them only focus on a single modality representation and learning. Though multi-sensors are considered in others, different modalities are represented in different spaces, which is bad for spatial consistency. Being different from them, GARF projects and aligns all modalities in polar BEV, which is beneficial to the design of the fusion model and adaptability to different sensor configurations.

## 2. Materials and Methods

### 2.1. Effectiveness Analysis of Cartesian and Polar Representations

**Figure 2:**
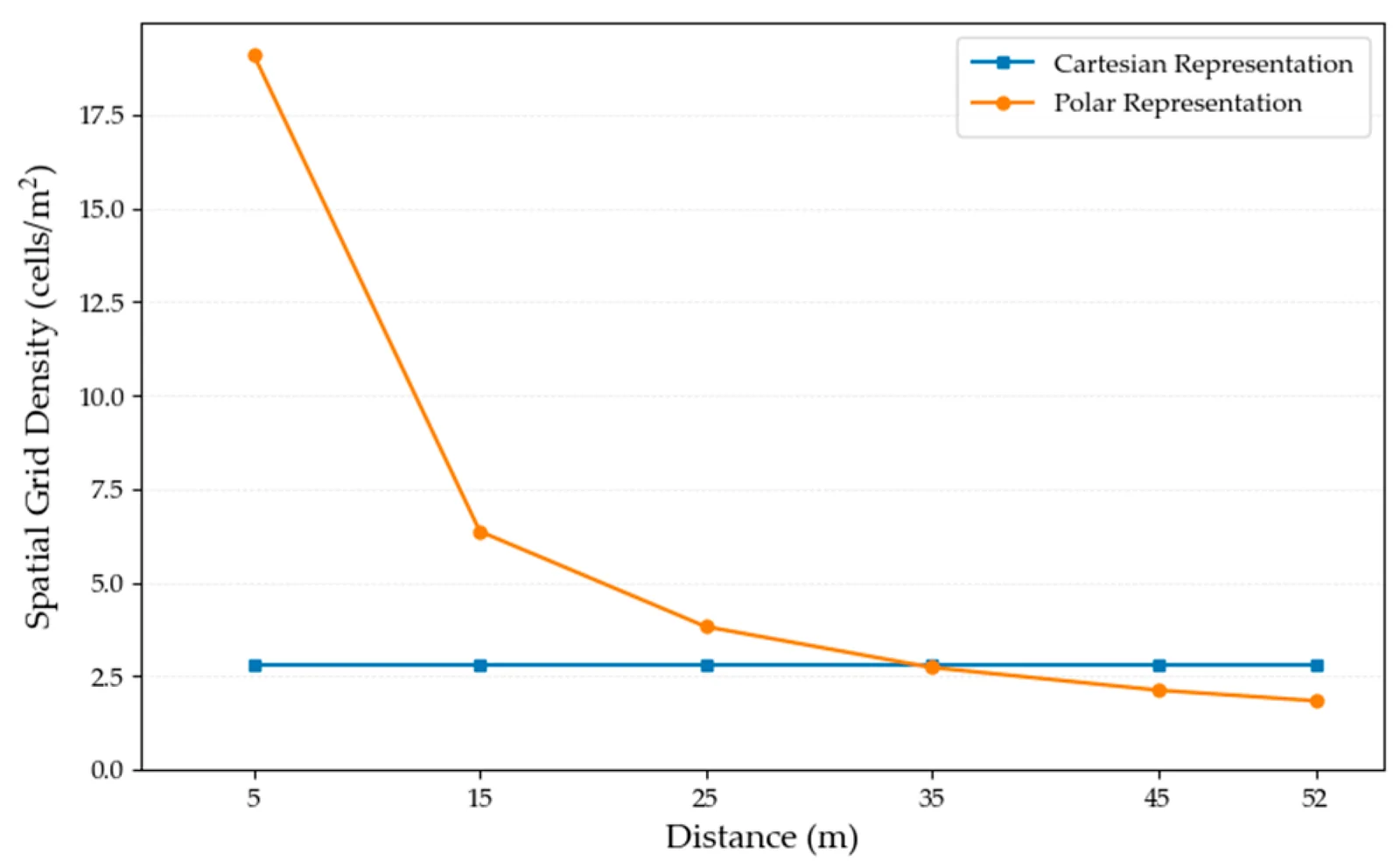
Comparison results of Cartesian and polar representations.

To show the advantage of polar BEV, the grid density is used to measure the effectiveness of different representations, and the result is shown in Figure 2. As the distance increases, the spatial resolution of the Cartesian representation remains unchanged, while that of the polar representation decreases. This shows that in the far region, the grid density of the polar representation becomes sparser, and less data are required. With similar resolution, the cell number can be reduced by about 50% by polar representation, which improves the computation efficiency greatly. On the other hand, according to the measurement mechanism of the camera and LiDAR, the distribution of the original measured data also satisfies this characteristic, i.e., dense in the near region and sparse in the far region. This implies that the effective information will not be lost by polar representation.

### 2.2. Overall Architecture

**Figure 3:**
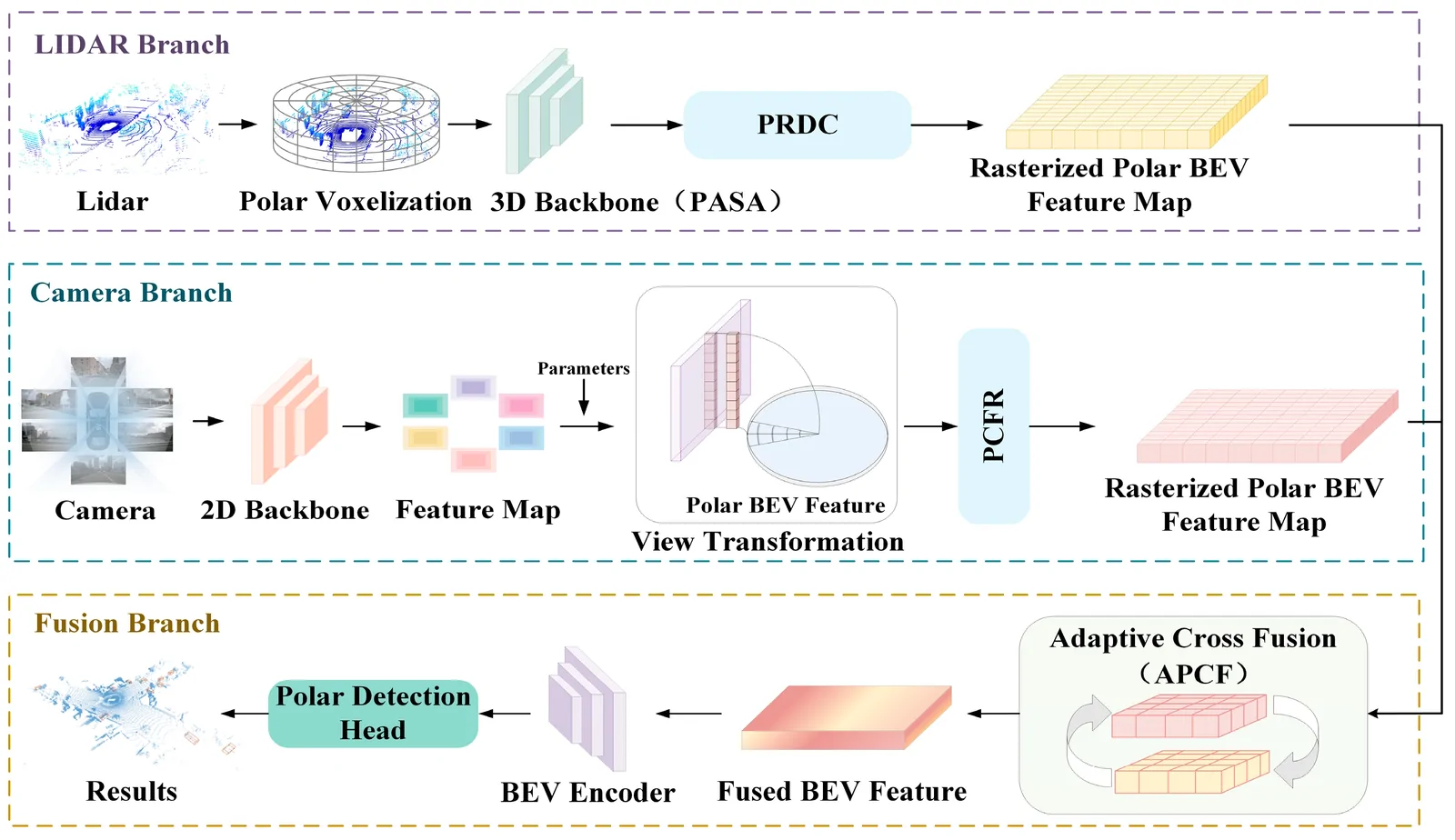
Framework of GARF.

The framework of GARF is shown in Figure 3, whose input is the synchronized multi-view images and LiDAR point clouds. Firstly, features are extracted for each modality separately, which are further aligned in the unified polar BEV space for fusion and object detection. In the LiDAR branch, the Polar Aware Asymmetric Sparse Backbone (PASA) and the Radial Conditioned Angular Recalibration module (PRDC) are designed to extract geometric features and reduce the distortion caused by polar mapping, respectively. In the camera branch, the uncertainty-guided view transformation and Polar Camera Feature Refinement (PCFR) are presented to extract and project image features to polar space robustly. In the polar BEV space, the Adaptive Polar Cross Fusion Module (APCF) is designed to fuse the features of the camera and LiDAR to predict the center, size, orientation, velocity, and 3D bounding box of the object by the polar detection head.

### 2.3. Polar Aware LiDAR Feature Extraction

As shown by the LiDAR branch in Figure 3, the point cloud is first transformed into polar coordinates $\left(\theta ,\rho ,z\right)$ and voxelized, and then sequentially processed by PASA and PRDC, which are designed as follows in detail.

**Figure 4:**
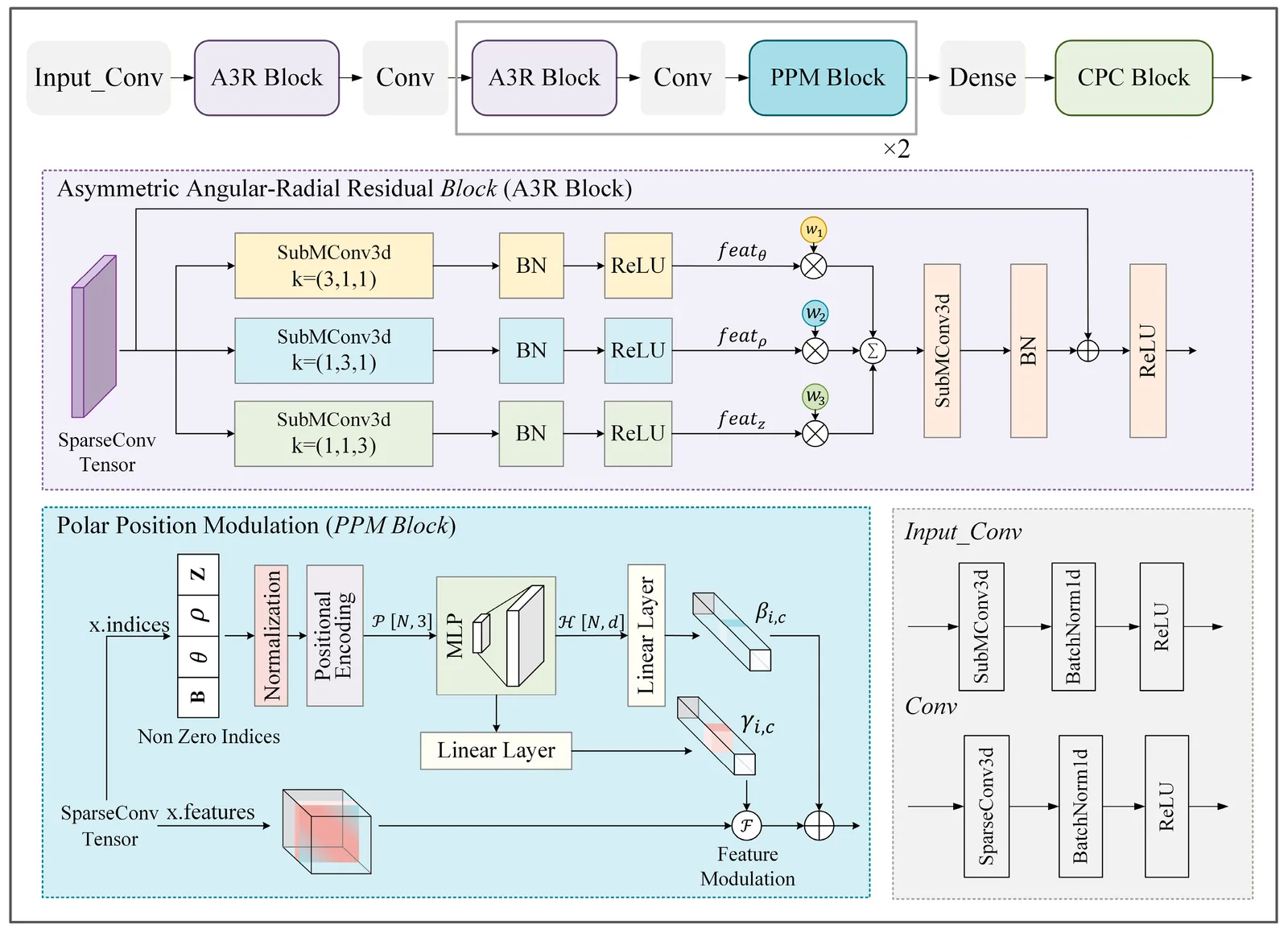
Architecture of polar-aware asymmetric sparse backbone.

**Polar Aware Asymmetric Sparse Backbone (PASA)**: Being different from the regular Cartesian grids, polar voxelization changes the spatial distribution of BEV features because of its asymmetric geometric characteristics. Directly adopting the existing backbone cannot fully exploit the advantages of polar representation. Although some studies have introduced geometry-aware modules to refine polar features, they act on the extracted features. Considering that the backbone is the foundation for learning geometric representations, we adopted the polar-aware adaptation in the baseline backbone. To better exploit the advantage of polar representation, PASA, as shown in Figure 4, is designed for the extraction of fine sparse 3D features. Compared with the baseline backbone, PASA is composed of three complementary sub-components: (a) asymmetric tri-axis residual (A3R) enhances directional feature learning to model spatial asymmetry; (b) Polar Position Modulation (PPM) has embedded coordinate-dependent geometric priors to capture position-dependent variations; (c) Circular Padding Calibration (CPC) ensures angular continuity across periodic boundaries. These interdependent components together improve the geometric modeling capability of sparse convolutional networks in polar space.

One challenge of polar space is the anisotropic geometric structure. Conventional cubic convolution uses a shared isotropic kernel and is not good at dealing with anisotropic geometric structure, and so it is replaced by A3R with an asymmetric tri-axis residual design, which enables heterogeneous feature extraction along different axes. With this axis-specific representation learning, it can model directional geometric structures while maintaining the efficiency of sparse convolutional operations. Another challenge arises from the non-uniform discretization of polar space. To deal with this, explicit polar positional priors are integrated into PPM for sparse feature learning. The polar position is encoded by the sparse voxel indices as follows:

(1) $$\begin{array}{c} {\tilde{\rho }}_{i}=\frac{i_{\rho }}{R_{dim}-1},\;\;{\tilde{\theta }}_{i}=\frac{2\pi i_{\theta }}{\Theta_{dim}-1}-\pi ,\;\;p_{i}=\left({\tilde{\rho }}_{i},\sin\left({\tilde{\theta }}_{i}\right),\cos\left({\tilde{\theta }}_{i}\right)\right). \end{array}$$

Then, it is further projected by a lightweight MLP to generate channel-wise modulation parameters $F_{i}^{\prime }$:

(2) $$\begin{array}{c} F_{i}^{\prime }=F_{i}\cdot \left(1+\gamma_{i}\right)+\beta_{i}. \end{array}$$

Being different from conventional positional encoding, PPM performs coordinate-conditioned feature modulation, which improves the adaptability of the network to spatial location.

In addition to the aforementioned two problems, there exists discontinuity along the angular dimension of polar BEV. The physical region near −π and π are adjacent, but the zero-padding of the convolution operator causes discontinuities at the boundary, which is bad for feature aggregation and spatial consistency. To ensure angular continuity, the following topology-aware circular padding is designed and integrated into CPC:

(3) $$\begin{array}{c} P=\left\lfloor \frac{K}{2}\right\rfloor , \end{array}$$

where K is the convolution kernel size and $\left\lfloor \cdot \right\rfloor$ denotes the floor operation. For a feature map $X_{BEV}\in R^{B\times C\times H\times W}$, its angular dimension is extended as follows:

(4) $$\begin{array}{c} X_{pad}\left[:,:,\theta^{\prime },:\right]=\left\{\begin{array}{ll} X_{BEV}[:,:,H-P+\theta^{\prime }], & if\;\theta^{\prime }<0\\ X_{BEV}[:,:,\theta^{\prime }], & if\;0\leq \theta^{\prime }<H\\ X_{BEV}[:,:,\theta^{\prime }-H], & if\;\theta^{\prime }\geq H \end{array}\right. \end{array}$$

By this, CPC ensures continuous aggregation and geometric consistency across the boundaries.

**Figure 5:**
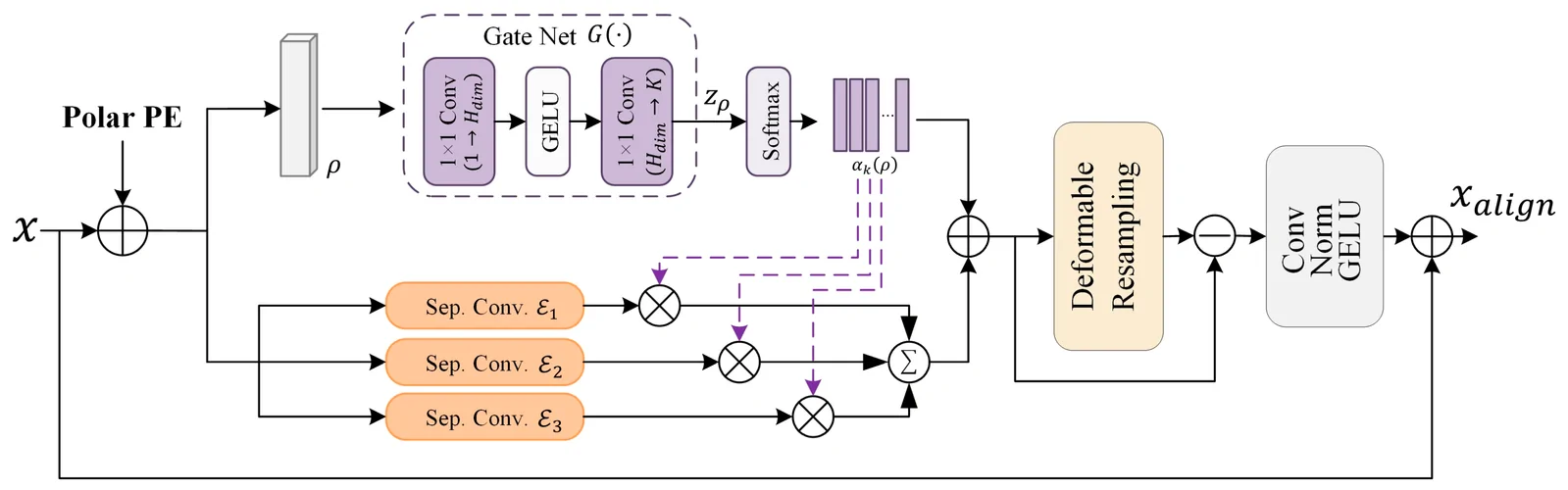
Architecture of polar re-alignment and distortion correction.

**Polar Re-alignment and Distortion Correction (PRDC)**: Although PASA improves polar-aware feature extraction, the arrayed polar BEV feature $X\in R^{B\times C\times H_{\theta }\times W_{\rho }}$ still suffers from location-dependent feature inconsistency caused by polar discretization. The representation of features with similar structures in physical space will become different at different radial distances and angular positions, which degrades the geometric consistency of polar BEV representation. Therefore, we propose a PRDC, shown in Figure 5, to recalibrate polar BEV features for better geometric consistency, which is beneficial to subsequent feature fusion.

As shown in Figure 5, PRDC is designed as a unified module to ensure geometric consistency in polar BEV space without adding new operations to extract features. It first uses polar positional priors to encode location-dependent geometric features. Then, these priors guide adaptive angular aggregation to compensate for distance-dependent distortion. Finally, geometric feature realignment further corrects spatial misalignment. These operations form a collaborative refinement process to attenuate the spatial distortion in polar BEV. The polar coordinate prior is defined as

(5) $$\begin{array}{c} P=\left[\rho_{norm},\;sin\theta ,cos\theta \right]\in R^{B\times 3\times H\times W}, \end{array}$$

where $\rho_{norm}$ is the normalized radial coordinate. Since the degree of geometric distortion varies with the location of the object, the following learnable mask is designed to adaptively control the injection strength of positional information:

(6) $$\begin{array}{c} X_{p}=X+\sigma \left(f_{m}\left(X\right)\right)\cdot f_{p}\left(P\right), \end{array}$$

where $f_{m}\left(\cdot \right)$ and $f_{p}\left(\cdot \right)$ are the projection functions. This design enables the positional priors to focus on ambiguous regions, while avoiding unnecessary perturbation on reliable features.

In polar space, the same angular kernel represents different receptive fields in physical space, since the area covered by a fixed angular interval increases with the radial distance. This causes inconsistent feature aggregation of surrounding objects at different ranges. To deal with this problem, multiple angular aggregation branches are designed as shown in Figure 5, which are further adaptively combined according to the radial distance. By this design, the angular receptive field can adapt to the actual physical space finely for better scale consistency of features in polar BEV. Though this distance-adaptive strategy is effective for large-scale distortions, local geometric misalignments after discretization still exist. The geometry-aware deformable resampling is adopted in PRDC to further improve the structural consistency. A lightweight network is used to estimate a learnable displacement field $\Delta G=\left(\Delta \theta ,\Delta \rho \right)$ and the aligned features are sampled through the following:

(7) $$\begin{array}{c} X_{d}=\mathrm{Sample}\left(X_{a},G+\Delta G\right). \end{array}$$

By adaptively correcting the location-dependent offset of features, PRDC finely ensures the structural consistency of object representation and successfully alleviates the distortion caused by polar discretization.

### 2.4. Uncertainty Guided Polar View Transformation

As shown by the camera branch in Figure 3, the Swin Transformer [51] backbone is used to extract image features, which are further sequentially processed by GADM and PCFR designed in detail as follows:

**Geometry-Aware Depth Estimation**: LSS has been widely used for view transformation due to its low computation consumption. Earlier designs generate pixel-wise depth distribution by a single depth prediction branch. Recently, some studies have introduced geometric information to improve depth estimation. To further deal with the negative influence of estimation uncertainty, besides the geometric prior, uncertainty-aware refinement is adopted in this study to design the Geometry-Aware Depth Estimation Module (GADM) for reliable depth estimation in polar view transformation.

**Figure 6:**
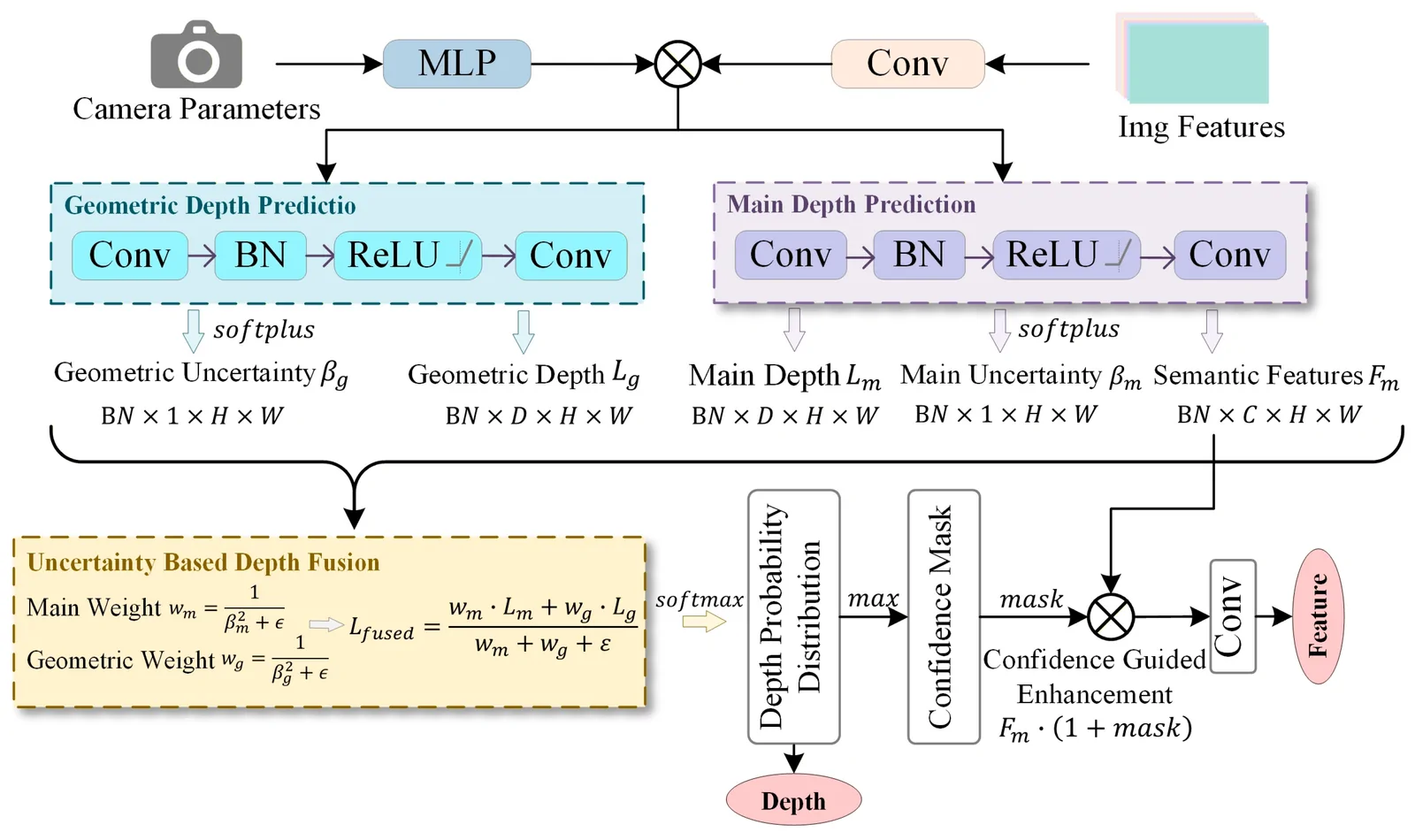
Architecture of geometry-aware depth estimation module.

As shown in Figure 6, compared with LSS and BEVDepth, GADM not only uses camera geometry for view transformation and depth enhancement, but also encodes intrinsic and extrinsic parameters and image augmentation transformation into a compact embedding to modulate image features before depth prediction. Then, it is further combined with the uncertainty-guided fusion. For conciseness, the vectorized intrinsic matrix K, extrinsic parameters E, and augmentation transformation M are concatenated into the geometry vector $\xi_{geo}$, whose dimension is 22. The geometry vector is encoded into a compact embedding and used to modulate image features before depth prediction, which improves the ability of the network to capture the geometric relationship between the image and physical space. Furthermore, a dual branch is designed in GADM, i.e., the main depth branch and geometry-aware auxiliary branch. Both predict the discrete depth distributions and uncertainties. The final result is obtained through uncertainty-guided fusion, whose weights are determined by the inverse of uncertainty. Then, the obtained 3D frustum points $p=\;\left(u,\;v,\;d\right)$ are projected into the coordinate of LiDAR using LSS [13], and finally BEVPoolv2 [52] is adopted to aggregate frustum features into the polar BEV to obtain the initial BEV representation of camera features.

**Polar Camera Feature Refinement (PCFR)**: There exist radial trailing artifacts in the generated camera features in the BEV because of the depth uncertainty and angular discontinuities at the −π/π boundary. To deal with its feature misalignment and degraded geometric consistency, the PCFR module is designed to refine camera features in the polar BEV. Firstly, the input feature F is decomposed into the fixed group and shifted group. The fixed group represents the original spatial layout, and the shifted group cyclically shifts along the angular dimension by the step size s:

(8) $$\begin{array}{c} {\hat{F}}_{shift}\left(h,\cdot \right)=F_{shift}\left(\left(h+s\right)\mathrm{mod}H,\cdot \right), \end{array}$$

The two features are then concatenated along the channel dimension to form $F_{mixed}$, which is partitioned into non-overlapping windows. Within each window, the following relative position multi-head self-attention is applied:

(9) $$\begin{array}{c} \mathrm{Attention}\left(Q,K,V\right)=\mathrm{Softmax}\left(\frac{QK^{T}}{\sqrt{d_{k}}}+B_{rel}\right)V, \end{array}$$

where Q, K, and V are obtained by linear projections, $d_{k}$ is the scaling factor, and $B_{rel}$ is the angular bias for relative position. In this way, the network efficiently models the angular continuity across the boundary.

To further suppress the radial trailing noise, a 1×K dilated convolution is adopted in PCFR to obtain the contextual feature $F_{ctx}$. With this, the model can identify effective features of objects. This can better distinguish compact object responses from elongated trailing artifacts caused by depth uncertainty. Based on the extracted context, the confidence mask is generated to adaptively suppress the noise:

(10) $$\begin{array}{c} G=\sigma (Conv_{1\times 1}(\delta (GN(Conv_{1\times 1}(F_{ctx}))))), \end{array}$$

where $\sigma \left(\cdot \right)$ is the sigmoid function, $\delta \left(\cdot \right)$ is GELU activation, and $GN\left(\cdot \right)$ is GroupNorm. The denoised feature is obtained by the following:

(11) $$\begin{array}{c} F_{out}=\mathrm{Conv}\left(F_{in}\cdot G\right)+F_{in}, \end{array}$$

where G is the confidence map. Finally, a lightweight recalibration layer is applied to enhance the semantic channel by rescaling the refined feature.

### 2.5. Polar BEV Feature Fusion

With the networks designed in Section 2.3 and Section 2.4, the features extracted from camera and LiDAR are projected and spatially aligned in the polar BEV, but they still differ in semantics. In this section, an adaptive fusion strategy, i.e., APCF, is developed for more effective interaction among cross-modal modalities.

**Figure 7:**
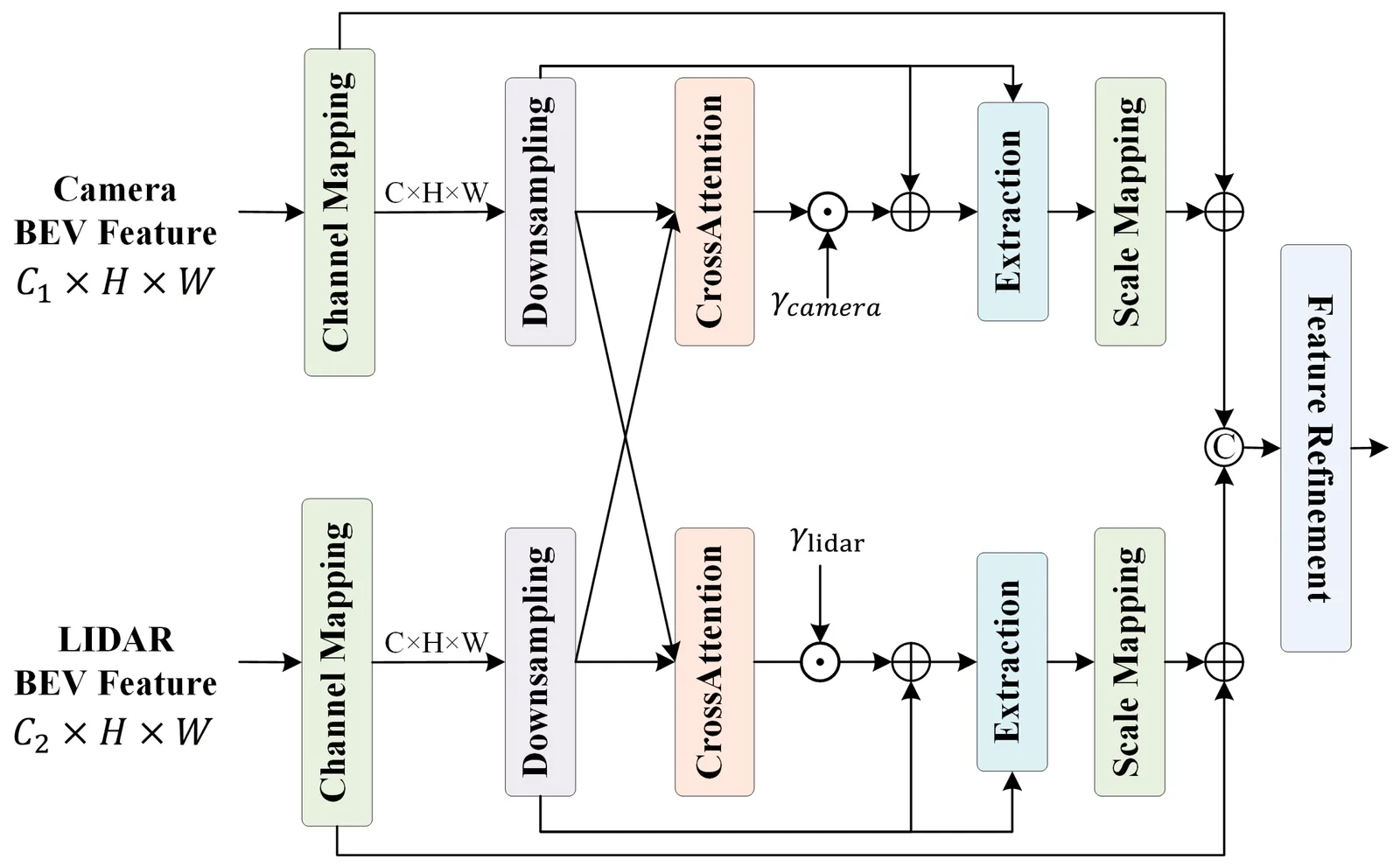
Architecture of adaptive polar cross fusion.

**Adaptive Polar Cross Fusion (APCF)**: As illustrated in Figure 7, APCF is designed to leverage the advantage of polar BEV representation by modeling the complementary relationship between camera and LiDAR features. It performs cross-modal interaction at coarse scale to capture complementary information, and then enhances high-resolution features through feature refinement.

With the camera features $F_{cam}\in R^{B\times C_{1}\times H\times W}$ and LiDAR features $F_{lidar}\in R^{B\times C_{2}\times H\times W}$, the learnable down-sampling is adopted to obtain a coarse semantic representation, which is further processed by the designed bidirectional cross attention mechanism with decomposed polar relative position biases. Taking the interaction from camera to LiDAR as an example, the query $Q_{cam}$ is generated from the camera branch, and the key $K_{lidar}$ and value $V_{lidar}$ are generated from the LiDAR branch. The cross-attention is obtained by the following:

(12) $$\begin{array}{c} \mathrm{CrossAttn}=\mathrm{Softmax}\left(\frac{Q_{cam}K_{lidar}^{T}}{\sqrt{d_{h}}}+B_{\theta }\left(\Delta \theta_{ij}\right)+B_{\rho }\left(\Delta \rho_{ij}\right)\right)V_{lidar}, \end{array}$$

where $d_{h}$ is the channel dimension of the attention head, $B_{\theta }$ and $B_{\rho }$ are learnable relative position biases along the angular and radial dimensions, respectively. Then, a learnable channel-wise scaling vector $\gamma_{cam}\in R^{D}$ is used to control the strength of cross-modal correction as follows:

(13) $$\begin{array}{c} {\tilde{F}}_{cam}^{c}=F_{cam}^{c}+\gamma_{cam}⊙\mathrm{CrossAttn}\left(F_{cam}^{c},F_{lidar}^{c}\right). \end{array}$$

With the bidirectional cross-modal interaction, the coarse features contain complementary global cues from different modalities. However, the spatial details may be lost if directly using these coarse features, which is critical to localization accuracy. For each modality $k\in \left\{cam,lidar\right\}$, the semantic increment is estimated by the following:

(14) $$\begin{array}{c} \Delta \Phi_{k}={\tilde{F}}_{k}^{c}-F_{k}^{c}. \end{array}$$

The correction is then upsampled and added to the original feature with high resolution as follows:

(15) $$\begin{array}{c} F_{k}^{fine}=F_{k}+{Upsample}_{bilinear}\left(\Delta \Phi_{k}\right) \end{array}.$$

Finally, the refined camera and LiDAR features are concatenated and processed by a lightweight convolutional head, where 1×1 convolution performs cross-modal channel mixing and 3×3 convolution further refines local spatial details.

**Polar Head**: In the polar BEV, the object is described by $\left(\theta ,\rho ,z,w,h,l,\alpha_{p},v_{\theta },v_{\rho }\right)$ instead of $\left(x,y,z,w,h,l,\alpha_{c},v_{x},v_{y}\right)$, widely used in Cartesian coordinates. Here $\left(x,y,z\right)$ denotes the object center, $\left(w,h,l\right)$ denotes its dimension, $\alpha_{c}$ is the yaw angle in Cartesian coordinates, and $\left(v_{x},v_{y}\right)$ denotes the velocity. The polar coordinate is defined as $\theta =\mathrm{atan}2\left(y,x\right)$, $\rho =\sqrt{x^{2}+y^{2}}$, $\alpha_{p}=\alpha_{c}-\theta$, $v_{\rho }$ and $v_{\theta }$ denote the radial and tangential velocity, respectively. To supervise the geometry center, an anisotropic Gaussian center map is designed in the polar feature space. Being different from Cartesian BEV, the angular and radial axes denote different physical meanings in polar BEV. Therefore, directly applying an isotropic Gaussian in the discrete index space cannot provide consistent physical supervision across different locations. In a local neighborhood around an object center $\left(\theta_{c},\rho_{c}\right)$, the squared Euclidean distance in polar coordinates can be approximated as $d^{2}\approx d_{\rho }^{2}+\rho_{c}^{2}d_{\theta }^{2}.$ This relation shows that angular and radial offsets should not be treated equally in the index space. Accordingly, our formulation converts both offsets into physical displacements and normalizes them using the angular and radial extents of the object. The resulting response is therefore aligned with the physical footprint of the object rather than the discrete structure of the polar grid.

For an object whose center is $\left(\theta_{c},\rho_{c}\right)$, the angular span is computed with circular difference as follows:

(16) $$\begin{array}{c} L_{\theta }=\rho_{c}\Delta \theta_{obj},\;L_{\rho }=\Delta \rho_{obj} \end{array}$$

where $\Delta \theta_{obj}$ and $\Delta \rho_{obj}$ are the angular and radial spans, respectively. The physical standard deviations are defined as follows:

(17) $$\begin{array}{c} \sigma_{\theta }^{phys}=\frac{L_{\theta }}{\gamma },\sigma_{\rho }^{phys}=\frac{L_{\rho }}{\gamma }, \end{array}$$

where γ is a constant and controls the effective range of the Gaussian distribution. A smaller γ leads to a sharper heatmap, which provides stricter supervision on center localization. On the contrary, a larger γ implies better tolerance to discretization error. In this study, it is set to 12 empirically. The physical displacement is approximated by the following:

(18) $$\begin{array}{c} d_{\theta }=\rho_{c}\delta_{\theta }s\Delta i_{\theta },d_{\rho }=\delta_{\rho }s\Delta i_{\rho }, \end{array}$$

where $\Delta i_{\theta }$ and $\Delta i_{\rho }$ are offsets of feature index, $\delta_{\theta }$ and $\delta_{\rho }$ are the polar BEV resolutions, and s is the downsampling factor. The anisotropic Gaussian center map is then defined in the physical space as follows:

(19) $$\begin{array}{c} G\left(\Delta i_{\theta },\Delta i_{\rho }\right)=\exp\left(-\frac{d_{\theta }^{2}}{2{\left(\sigma_{\theta }^{phys}\right)}^{2}}-\frac{d_{\rho }^{2}}{2{\left(\sigma_{\rho }^{phys}\right)}^{2}}\right). \end{array}$$

## 3. Results

Given synchronized multi-view images I, LiDAR point cloud P, camera calibration parameters C, and 3D annotations Y, the objective is to learn a multimodal detector $f_{\Theta }$ that predicts 3D objects $\hat{Y}$ in the ego coordinate system as

(20) $$\begin{array}{c} \hat{Y}=f_{\Theta }(I,P,C). \end{array}$$

GARF constructs camera and LiDAR features in a unified polar BEV space, followed by geometry-aware feature refinement and multimodal fusion. The parameters of $f_{\Theta }$ are optimized by

(21) $$\Theta *=arg\;\underset{\Theta }{min}\frac{1}{N}\sum_{i=1}^{N}L_{det}\left(f_{\Theta }(I_{i},P_{i},C_{i}),Y_{i}\right).$$

where $L_{det}$ is the loss function used for object classification and 3D bounding box regression.

The resolution of the input image is 256 × 704, whose 2D features are extracted by Swin Transformer, and the point cloud of LiDAR is encoded by hard voxelization. Then, a 128 × 64 polar BEV grid is obtained by combining 80 channel camera features and 256 channel LiDAR features. The radial and angular resolutions are selected by considering the trade-off between detection accuracy and computational efficiency. The range of polar BEV along the radial and angular dimensions is 0 to 72.4 m and −π to π, respectively. The polar grid represents locations by radial and angular coordinates. In PRDC, the offset prediction network consists of two 1 × 1 convolutions with the dimensions 256 → 64 → 2. The parameter of the final convolution is initialized as zero, and only radial offsets are predicted, with a maximum displacement of 6 pixels, an initial offset scale of 2 pixels, and a residual scaling factor initialized to $10^{-2}$. PCFR uses the four-head angular window attention with a window size of 8 and a cyclic shift of 4 bins, a 1 × 7 radial depthwise convolution, an 80 → 40 → 80 gated suppression network, and an FFN with an expansion dimension of 320. All models are trained with AdamW using a weight decay of 0.01, an initial learning rate of $5.0\times 10^{-5}$, cosine annealing, and a total batch size of 8. For the fusion model, the LiDAR branch is first pretrained for 20 epochs, followed by joint optimization of the camera and LiDAR branches for another 6 epochs without freezing any parameters. All experiments are conducted on two NVIDIA RTX A6000 GPUs (NVIDIA Corporation, Santa Clara, CA, USA).

### 3.1. Comparison with Representative Methods

To evaluate and validate the effectiveness of the proposed method, several comparative tests with several representative methods have been conducted on nuScenes.

As shown in Table 2, GARF achieves 73.1% mAP and 75.2% NDS, and compared with BEVFusion, the performance is improved by 2.9% mAP and 2.3% NDS. On the validation set, GARF achieves 71.8% mAP and 73.7% NDS. Overall, GARF improves all metrics over the baseline, including mATE, mASE, mAOE, mAVE, and mAAE. This shows that the proposed polar BEV representation benefits not only object classification and recall, but also localization, scale estimation, orientation prediction, velocity estimation, and attribute recognition. Moreover, the dimension of the BEV grid used by GARF is only 128×64, which is much less than 128×128 used by the baseline.

### 3.2. Ablation Studies

To further analyze the contribution of each module, ablation studies are conducted in this section for the camera branch, LiDAR branch, and the fusion branch, respectively.

#### 3.2.1. Analysis of LiDAR Branch

Compared with the baseline, PASA and PRDC are presented in this study. All experiments are conducted with the same training configuration. As shown in Table 3, the computation efficiency is improved by directly replacing Cartesian BEV with polar BEV, but the accuracy becomes worse. The polar baseline increases FPS from 10.5 to 12.9 and reduces the memory usage from 18,802 MiB to 13,500 MiB, while mAP and NDS decrease from 0.641 and 0.689 to 0.627 and 0.674, respectively. This implies that polar representation is computationally efficient, but polar voxelization causes non-uniformity of features and geometric distortions, which is bad for detection performance.

With the adoption of the newly designed modules, the detection performance is improved. Among them, PASA improves mAP and NDS to 0.638 and 0.681. This shows that the direction-aware extraction method for sparse features can describe the anisotropic structure of polar voxels finely. PRDC improves the baseline by correcting geometric distortions. When PASA and PRDC are both used, GARF achieves the best performance, i.e., 0.648 mAP and 0.697 NDS, while having higher FPS and lower memory usage.

#### 3.2.2. Analysis of Camera Branch

In this section, GADM and PCFR in the camera branch are evaluated, and the results are shown in Table 4. Compared with Cartesian BEV, the performance is improved by using polar BEV overall. The polar baseline improves mAP from 0.358 to 0.363 and NDS from 0.417 to 0.421, meanwhile increasing FPS from 6.7 to 8.2 and reducing memory usage from 40,492 MiB to 21,366 MiB. This shows that polar BEV is more suitable for the view transformation of the camera, since it is more compact and matches the perspective geometry better.

The proposed GADM and PCFR further improve the performance. GADM increases mAP and NDS to 0.374 and 0.431, respectively. This shows that geometry priors and uncertainty-guided fusion can improve the robustness of depth estimation during view transformation. PCFR also improves the performance of polar baseline, which shows its ability to refine features in polar BEV by reducing radial noise and enhancing angular continuity. When GADM and PCFR are used, GARF achieves the best performance with 0.381 mAP and 0.442 NDS.

#### 3.2.3. Analysis of Fusion Branch

This section analyzes the effectiveness of the fusion branch, and the results are shown in Table 5. It can be found that both accuracy and efficiency are improved by replacing Cartesian BEV with polar BEV. Compared with the Cartesian baseline, the polar baseline improves mAP from 0.685 to 0.696 and NDS from 0.714 to 0.721, respectively, while increasing FPS from 7.1 to 9.3 and reducing memory usage from 41,114 MiB to 31,702 MiB. This indicates that polar BEV provides a more compact representation for multi-modalities and matches the sensing geometry better. The improvement by APCF shows that the explicit cross interaction in polar BEV effectively enhances feature complementarity between camera and LiDAR. Compared with the polar baseline, the anisotropic Gaussian center response map increases mAP and NDS to 0.701 and 0.726, respectively. This shows that the center supervision considering geometry consistency is beneficial to object localization, since angular and radial dimensions have different physical meanings in polar BEV. When APCF and the anisotropic Gaussian center response map are both used, GARF achieves the best performance with 0.718 mAP and 0.737 NDS, while having higher FPS and lower memory usage.

To further analyze the fusion strategy, APCF has been further compared with other fusion methods in Table 6. The main advantage of APCF is the joint design of geometry-guided interaction and residual refinement with high resolution. The former improves global semantic alignment between camera and LiDAR, and the latter reduces the loss of location details. Though the computation cost of the baseline is low, its detection performance is worse. The convolution-based fusion can improve local feature integration, but it cannot model long-range dependency and global relations between cross modalities. The attention-based fusion improves accuracy, but needs more memory usage and, accordingly, lower inference speed. APCF achieves the best mAP and NDS, while maintaining acceptable FPS and memory usage. This shows that APCF makes a good balance between detection performance and computation efficiency by performing cross-attention on down-sampled polar features and injecting the learned residuals back into the high-resolution features.

#### 3.2.4. Analysis of BEV Resolution

The resolution of the BEV feature directly determines the representation granularity of objects. A higher resolution can better represent the information of small objects and boundary details, which is beneficial to localization accuracy. However, this also increases computational cost and memory usage, leading to lower inference speed. Table 7 shows the results under different resolutions. As the resolution increases, the detection performance improves rapidly in the earlier stage, while the inference speed decreases. When the resolution increases from 120×60 to 180×90, mAP and NDS improve from 0.701 and 0.723 to 0.718 and 0.737, respectively, and FPS decreases from 14.1 to 8.9. Comparing 180×180 and 360×90 with the default 180×90, it is found that increasing the radial or angular resolution separately is not beneficial to the performance significantly. This implies that the angular and radial resolutions should be balanced by considering both accuracy and speed.

#### 3.2.5. Analysis of Detection Performance

To further analyze the influence of polar BEV on the detection of different types of objects, AP is shown in Table 8 by comparing with BevFusion. It is found that GARF improves AP for all categories. For small objects such as pedestrians, motorcycles, bicycles, and traffic cones, the improvement is mainly from the denser sampling in the near range of polar BEV, which reduces quantization error of the grid.

To further evaluate the robustness of GARF, it is tested under different environmental conditions, and the results are shown in Table 9. The results show that GARF is better than the baseline under all conditions. Comparatively, the improvement under rainy scenes is the least, and the best under night scenes. Overall, GARF provides more effective spatial representation and stronger cross-modal feature integration abilities.

**Figure 8:**
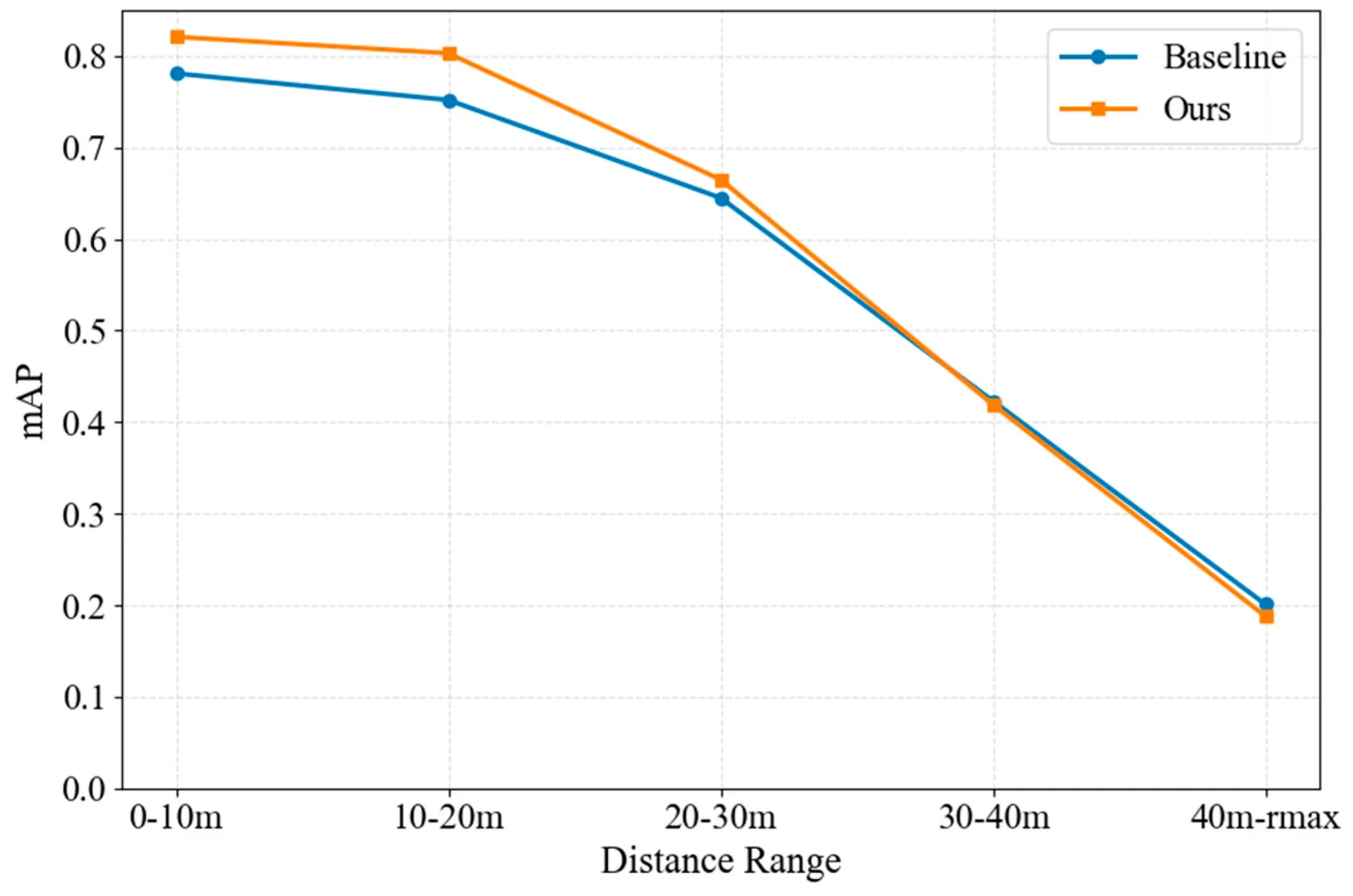
mAP results under different distances.

The detection performance under different distances is shown in Figure 8 comparatively. Overall, both show a clear drop in mAP as the distance increases, since for distant objects, their LiDAR points and image pixels are sparser. Compared with the baseline, GARF achieves more stable and better performance in both near and middle-distance ranges. At far distances, the improvement in mAP becomes smaller and even slightly decreases. Besides mPA, GARF also has the advantage in inference speed and memory usage, which shows that GARF provides a favorable trade-off between detection performance and computation efficiency.

#### 3.2.6. Qualitative Results

**Figure 9:**
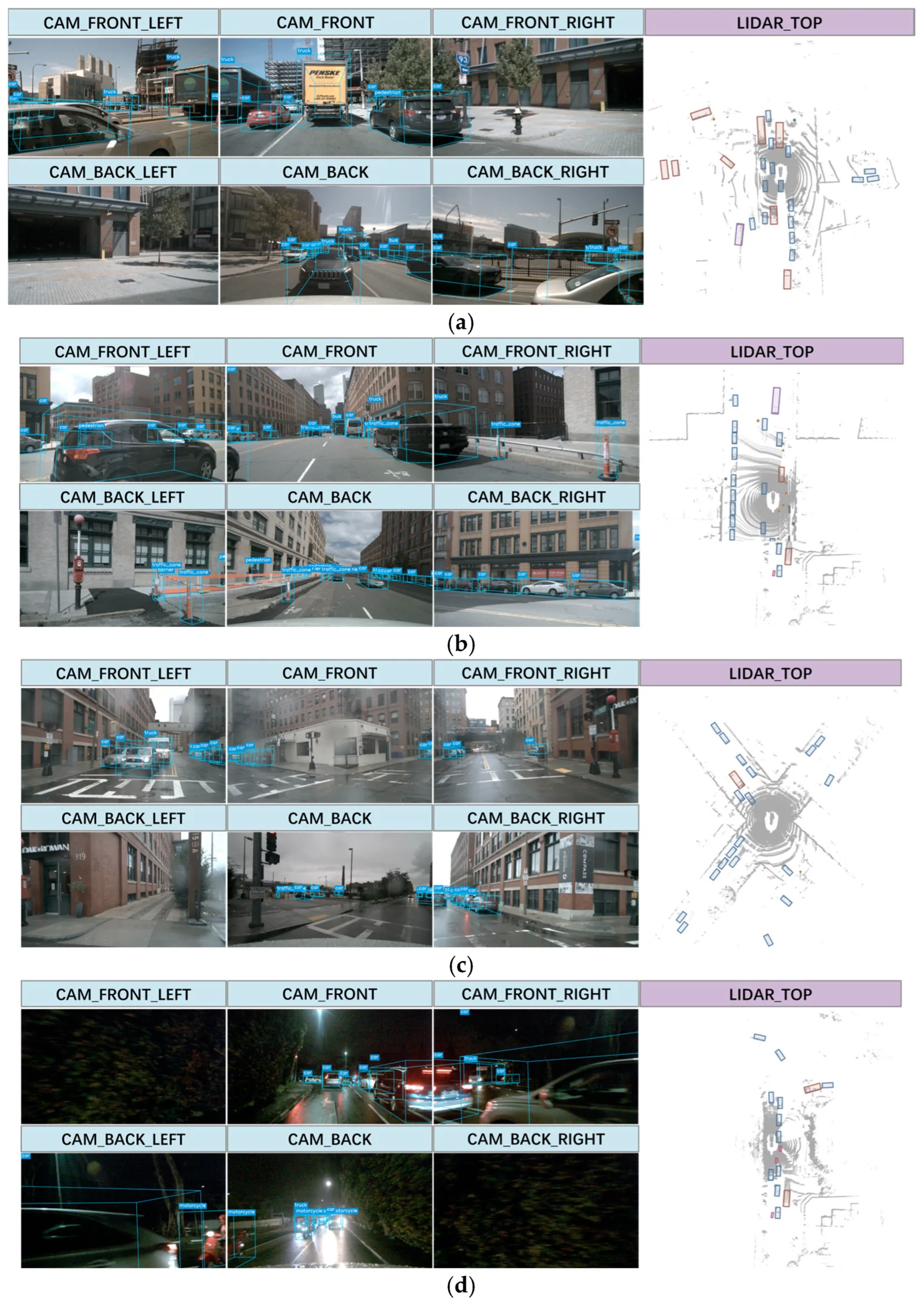
Qualitative results of nuScenes under different environmental conditions: (**a**) sunny, (**b**) daytime, (**c**) rainy, and (**d**) night.

To provide a more intuitive evaluation of GARF, some specific detection results are shown in Figure 9, where different colors denote object categories. The visualization results show that GARF can accomplish 3D detection under different driving scenes, including sunny, daytime, rainy, and night scenarios. In the camera views, the predicted boxes match the object regions. In the BEV views, the results clearly show the position, orientation, and spatial layout of surrounding objects.

## 4. Discussion

GARF provides a general and flexible framework to fuse multimodal sensors by polar BEV to achieve a good balance between detection accuracy and computation efficiency. The main motivation is to organize and refine BEV features according to the observation geometry of the sensor, which can also be extended to other BEV-based perception frameworks. For the LiDAR branch, the polar-aware feature extraction is suitable for radial scanning data and non-uniform points. For the camera branch, the uncertainty-guided view transformation can improve the accuracy and robustness of transmission from image to polar BEV. For multimodal fusion, the proposed strategy provides a general way to align texture and geometric cues in the shared polar BEV space.

The experimental results show that polar BEV provides a more consistent representation of the sensing geometry of cameras and LiDAR by organizing their spatial features along the radial and angular dimensions. Compared with Cartesian BEV, it represents the spatial distribution of objects in a more geometry-aware manner, which is especially suitable for near and middle range regions, where objects are denser and critical for autonomous driving.

GARF is more effective in near and middle range regions. This is mainly due to the sparsity of LiDAR points and image pixels of distant objects. Moreover, in highly cluttered urban scenes, multiple objects may also fall into neighboring angular bins, increasing feature overlap and reducing object separability, especially when they are aligned along similar viewing directions. These limitations indicate that a fixed polar grid may become less effective for long-range or densely cluttered perception. More flexible representation, such as adaptive radial partitioning, dynamic angular resolution, or continuous spatial modeling, may further improve long-range detection ability.

The evaluation and validation of GARF are conducted on nuScenes in this study. NuScenes contains diverse urban scenes, but it cannot cover all complex long-tail cases, such as severe weather, dense traffic, rare object layouts, and domain shifts across cities or sensor platforms. Changes in perception configuration, such as sensing range, field of view, sensor placement, and point density, influence the transformation between the fixed polar grid and physical space. Re-training and calibration are necessary to adapt to the new configuration.

To evaluate GARF more comprehensively, in the future more critical conditions and some open questions can be considered including: (a) test with more open datasets, such as Waymo Open Dataset and ONCE; (b) test under independent samples with different mean and standard deviations; (c) test by deploying in real vehicle with different configurations of perception system; (d) more metrics for throughout evaluation; (e) and systematic sensitivity analysis about the key parameters, i.e., polar discretization, Gaussian parameters, receptive fields, and module configuration.

## 5. Conclusions

In this study, GARF is designed for multi-modal 3D object detection. Instead of Cartesian BEV space, a unified polar BEV representation for both camera and LiDAR features is designed for GARF. In the camera branch, the uncertainty-guided polar view transformation is presented to improve the estimation reliability of depth and refine features in polar BEV. In the LiDAR branch, two specific modules are designed to achieve polar-aware extraction of sparse features and correct geometric distortion caused by polar voxelization. In the fusion stage, the region-aware fusion strategy and the anisotropic Gaussian center response map are presented to enable cross-interaction of multi-modalities and consistent supervision of the geometric center in polar BEV.

Several comparative experiments carried out on nuScenes demonstrate the effectiveness of GARF. Compared with the baselines, GARF achieves competitive detection performance, while reducing memory usage and improving inference efficiency. Ablation studies further show the contribution of each component. Overall, GARF can strike a favorable balance between detection accuracy and computational cost.

## Figures and Tables

**Table 1:** Comparison between GARF and other 3D detection methods based on polar BEV.

Method	Modality	Characteristic	Difference from GARF
PolarStream [44]	L	Sector-wise perception	No geometry-aware feature alignment
PARTNER [45]	L	Correction of feature distortion	Feature alignment after polar representation, while GARF adopts polar-aware feature extraction in backbone.
PolarFormer [46]	C	Irregular polar BEV learning	Transformer-based learning, whereas GARF optimizes geometry-aware in LSS.
PolarBEVDet [47]	C	Polar transformation and temporal fusion.	No consideration of depth uncertainty
PolarGFusion3D [49]	CLR	Polar graph-based multimodal fusion	3D polar voxel features are directly fused by graph aggregation, whereas GARF optimizes features for specific modality before adaptive fusion.
PolarFusion [50]	CL	Polar region-based cross-modal fusion.	Camera and LiDAR are represented in image and polar spaces, respectively, whereas different modalities are projected and aligned on polar BEV in GARF for better consistency and adaptability.

L, C, and R denote LiDAR, camera, and radar modalities.

**Table 2:** Comparative results of nuScenes.

Method	Test Set							Validation Set	
	mAP↑	NDS↑	mATE↓	mASE↓	mAOE↓	mAVE↓	mAAE↓	mAP↑	NDS↑
UVTR [53]	0.671	0.711	0.306	0.245	0.351	0.225	0.124	0.654	0.703
TranFusion [6]	0.689	0.717	0.259	0.243	0.329	0.288	0.127	0.675	0.713
FTUR3D [54]	0.694	0.721	0.284	0.241	0.310	0.300	0.120	0.642	0.680
MMFUSION [55]	0.642	0.694	0.302	0.249	0.353	0.240	0.120	0.654	0.697
CMT [56]	0.704	0.730	0.299	0.241	0.323	0.240	0.112	0.694	0.719
MFT [37]	0.700	0.719	0.274	0.261	0.291	0.287	0.192	0.686	0.717
EA-LSS [57]	0.722	0.744	0.247	0.237	0.304	0.250	0.133	0.712	0.731
BEVFusion [7] *	0.702	0.729	0.261	0.239	0.329	0.260	0.137	0.685	0.714
GARF	0.731	0.752	0.239	0.221	0.311	0.245	0.119	0.718	0.737

All results are obtained under the same setting without temporal information. The symbol “∗” denotes the baseline, ↑ and ↓ indicates which direction means better results.

**Table 3:** Ablation study results of LiDAR branch.

Method	Coordinate	PASA	PRDC	mAP↑	NDS↑	FPS↑	Memory/GPU
Baseline	Cartesian	×	×	0.641	0.689	10.5	18,802 MiB
Baseline	Polar	×	×	0.627	0.674	12.9	13,500 MiB
Baseline	Polar	√	×	0.638	0.681	12.4	13,970 MiB
Baseline	Polar	×	√	0.633	0.679	12.6	15,426 MiB
GAPF	Polar	√	√	0.648	0.697	12.2	15,774 MiB

√ and × indicate whether the component is used or not, respectively. ↑ indicates that a higher value is better. Memory/GPU means used memory during training.

**Table 4:** Ablation study results of camera branch on nuScenes.

Method	Coordinate	GADM	PRFR	mAP↑	NDS↑	FPS↑	Memory/GPU
Baseline	Cartesian	×	×	0.358	0.417	6.7	40,492 MiB
Baseline	Polar	×	×	0.363	0.421	8.2	21,366 MiB
Baseline	Polar	√	×	0.374	0.431	8.1	22,388 MiB
Baseline	Polar	×	√	0.369	0.424	7.8	21,426 MiB
GAPF	Polar	√	√	0.381	0.442	7.7	22,390 MiB

√ and × indicate whether the component is used or not, respectively. ↑ indicates that a higher value is better.

**Table 5:** Ablation study results of fusion branch on nuScenes.

Method	Coordinate	APCF	AGH	mAP↑	NDS↑	FPS↑	Memory/GPU
Baseline	Cartesian	×	×	0.685	0.714	7.1	41114 MiB
Baseline	Polar	×	×	0.696	0.721	9.3	31702 MiB
Baseline	Polar	√	×	0.709	0.729	8.9	33346 MiB
Baseline	Polar	×	√	0.701	0.726	9.2	31702 MiB
GAPF	Polar	√	√	0.718	0.737	8.9	33346 MiB

√ and × indicate whether the component is used or not, respectively. ↑ indicates that a higher value is better. AGH denotes the anisotropic Gaussian center response map, and Memory/GPU denotes the used memory during training.

**Table 6:** Performance comparison between different fusion strategies.

Fusion Strategy	Description	mAP↑	NDS↑	FPS↑	Memory/GPU
Concat [7] *	Channel concatenation	0.696	0.721	9.3	31,702 MiB
UniBEVFusion [35]	Mainly convolution-based	0.701	0.723	9.1	32,457 MiB
MapFusion [38]	Mainly attention-based	0.707	0.726	8.6	36,127 MiB
APCF	Ours	0.709	0.729	8.9	33,346 MiB

* denotes the baseline, ↑ indicates that a higher value is better, and Memory/GPU denotes the used memory during training.

**Table 7:** Ablation study on BEV resolution on nuScenes.

Coordinate	Voxel Grid Size	BEV Feature Resolution	mAP↑	NDS↑	FPS↑
Polar	960×480	120×60	0.701	0.723	14.1
Polar	1440×720	180×90	**0.718**	**0.737**	**8.9**
Polar	1920×960	240×120	0.721	0.742	4.8
Polar	1440×1440	180×180	0.720	0.740	4.4
Polar	2880×720	360×90	0.721	0.740	4.3

↑ indicates that a higher value is better, Bold indicates the default BEV resolution.

**Table 8:** Detection performance of different categories.

Method	CAR	TRUCK	BUS	TRAILER	CONST.	PEDEST.	MOTOR.	BICYCLE	TRAFF.	BARRIER
BevFusion *	0.884	0.614	0.718	0.528	0.337	0.881	0.752	0.556	0.827	0.754
Ours	0.890	0.660	0.758	0.584	0.385	0.890	0.771	0.576	0.840	0.826

The symbol “*’’ denotes the baseline, and “Const.’’, “Pedest.’’, “Motor.’’, and “Traff.’’ denote construction vehicle, pedestrian, motorcycle, and traffic cone, respectively.

**Table 9:** mAP results under different environmental conditions.

Method	Sunny	Rainy	Day	Night
Baseline	0.682	0.699	0.685	0.428
GARF	0.716	0.721	0.718	0.524

## Data Availability

The original contributions presented in this study are included in the article. Further inquiries can be directed to the corresponding author.
